# Supplementary material for: Prolonged length of stays and its associated factors among pediatric surgical patients in Oromia, Central part of Ethiopia: A cross-sectional study
Source: PLoS One. 2025 Sep 10;20(9):e0330034. doi: 10.1371/journal.pone.0330034 (PMC12422484; doi:10.1371/journal.pone.0330034)
Supplement: Attachment — Submitted filename: PLOS one_Comments.pdf [file pone.0330034.s001.pdf]

Decision : Major revision

Comments:

Thank you for the opportunity to review this manuscript, which focuses on the prolonged length of stay among pediatric surgical patients in two hospitals in Ethiopia. I have outlined my feedback as follows:

General Comments:

There are various grammatical, typographical, and coherence issues throughout the document that warrant revision and reorganization of the paragraphs for better clarity and flow.

1. Title:

I suggest refining the title as:

"Prolonged Length of Hospital Stays and Its Associated Factors Among Pediatric Surgical Patients Admitted to Two Teaching Hospitals in Central Ethiopia: A Cross-Sectional Study."

2. Background:

- The authors state that no previous study has been conducted on this subject in the study setting. However, this argument appears weak as a justification for conducting the study.
- Additionally, various factors influencing the length of hospital stays are mentioned in the background section, and these overlap with the current study's findings. This raises concerns about the originality and potential contribution of the paper to the existing literature.
- I recommend clearly articulating the research gap and providing a strong justification for why this study is necessary.

3. Methodology:

- The authors mention the use of a simple random sampling technique to recruit participants. However, considering the prospective sampling of patients admitted at various times, it is unclear how the sampling frame was established and how computer-generated random sampling was conducted.
- **Outcome Variable:**
  - It is unclear why the 75th percentile was chosen as the cutoff point for defining prolonged stays.
  - The reference to the 75th percentile under the "Data Processing and Analysis" section needs clarification. Since this metric is based on the length of stay for all participants, its applicability to similar contexts may raise concerns about transferability.
- "No Risk" and "At Risk" Variables:
  - The classification of these variables is unclear and requires further explanation, along with appropriate citations.

- Translation of Tools:

- The translation process for the data collection tools is mentioned twice under the "Data Collection Method" and "Data Quality Control" sections. This redundancy should be addressed.

- Data Processing and Analysis:

- Readers may benefit from a clearer explanation of how normality and other assumptions for binary logistic regression were addressed. While multicollinearity is mentioned, further elaboration would enhance clarity.
- There is a duplicate statement about the assessment of the model's goodness of fit using the Hosmer and Lemeshow test, and the multicollinearity evaluation using the Variance Inflation Factor (VIF). Streamlining this information would improve readability.

4. Results:

- This section requires significant revision for grammar and coherence to improve its readability and clarity.

5. Discussion:

- The discussion primarily compares and contrasts the current findings with previous studies, providing justifications for similarities and differences.
- However, it is important to include practical recommendations for practice, policy, and research, particularly in the context of the Ethiopian healthcare system.
