# Supplementary material for: Prolonged length of stays and its associated factors among pediatric surgical patients in Oromia, Central part of Ethiopia: A cross-sectional study
Source: PLoS One. 2025 Sep 10;20(9):e0330034. doi: 10.1371/journal.pone.0330034 (PMC12422484; doi:10.1371/journal.pone.0330034)
Supplement: Attachment — Submitted filename: point by point response for Plos one(LOS pedi surgery).pdf [file pone.0330034.s002.pdf]

Title: **Prolonged Length of Hospital Stays and its Associated Factors among Pediatric Surgical Patients Admitted in Adama comprehensive specialized hospital and Asella Referral and Teaching Hospital, Oromia, Ethiopia: A cross-sectional study**

From: Authors

To: PLOS ONE

Version: 1

Point -by-point response by authors

Sections	Independent Review Report		Response to comments by Authors
	Reviewer #1		
General comments	There are various grammatical, typographical, and coherence issues throughout the document that warrant revision and reorganization of the paragraphs for better clarity and flow.		Dear respected reviewer, we thank you for your genuine comments. The comments are helpful to increase the quality our study.
Title	I suggest refining the title as: "Prolonged Length of Hospital Stays and Its Associated Factors Among Pediatric Surgical Patients Admitted to Two Teaching Hospitals in Central Ethiopia: A Cross-Sectional Study."		Thank you for your comments, refining based on your recommendation
Background	The authors state that no previous study has been conducted on this subject in the study setting. However, this argument appears weak as a justification for conducting the study.<br>- Additionally, various factors influencing the length of hospital stays are mentioned in the background section, and these overlap with the current study's findings. This raises concerns about the originality and potential contribution of the paper to the existing literature.<br>- I recommend clearly articulating the research gap		-Thank you for your comments, we have revised it accordingly.<br><br>-The various factors mentioned in the introduction part of the manuscript is based the existing literatures even outside the country just for comparison.<br><br>-Thank you, revised!

	and providing a strong justification for why this study is necessary.<br>…		
Methods	The authors mention the use of a simple random sampling technique to recruit participants. However, considering the prospective sampling of patients admitted at various times, it is unclear how the sampling frame was established and how computer-generated random sampling was conducted.<br>Outcome Variable:<br>- It is unclear why the 75th percentile was chosen as the cutoff point for defining prolonged stays.<br>- The reference to the 75th percentile under the "Data Processing and Analysis" section needs clarification. Since this metric is based on the length of stay for all participants, its applicability to similar contexts may raise concerns about transferability.<br>- "No Risk" and "At Risk" Variables:<br>- The classification of these variables is unclear and requires further explanation, along with appropriate citations.<br>- Translation of Tools:<br>- The translation process for the data collection tools is mentioned twice under the "Data Collection Method" and "Data Quality Control" sections. This redundancy should be addressed.<br>- Data Processing and Analysis:<br>- Readers may benefit from a clearer explanation of how normality and other assumptions for binary logistic regression were addressed. While multicollinearity is mentioned, further elaboration would enhance clarity.<br>- There is a duplicate statement about the assessment of the model's goodness of fit using the		-Thank you for your insight, this is due to editorial problem face to face interview did not conducted rather this is a one year study retrieved from February 1, 2023, to March 30 with the data extraction period of one month(April 1- 30, 2024). See the revised manuscript on the abstract section.<br><br>-Thank you, revised based on the comment<br><br>-Thank you, revised based on the comment<br><br>- Thank you ….Distribution of data were checked by shapiro-wilk and Kolmogorov tests(for p-value greater than 0.05,the data were considered as normally distributed) and, revised based on the comment

	Hosmer and Lemeshow test, and the multicollinearity evaluation using the Variance Inflation Factor (VIF). Streamlining this information would improve readability.		-Thank you, revised based on the comment
Result	This section requires significant revision for grammar and coherence to improve its readability and clarity.		-Thank you, revised based on the comment
Discussion	The discussion primarily compares and contrasts the current findings with previous studies, providing justifications for similarities and differences.<br>- However, it is important to include practical recommendations for practice, policy, and research, particularly in the context of the Ethiopian healthcare system.		Thank you, revised based on the comment
	Reviewer #2:		
General comments	I appreciate you, editor, asking me to review the manuscript "Prolonged Length of Hospital Stays and its Associated Factors Among Paediatric Surgical Patients Admitted in Adama Hospital Medical College and Asella Referral and Teaching Hospital, Oromia, Ethiopia: A cross-sectional study based on logistic regression analysis." The study will significantly impact paediatric surgical patients' management, and the length of hospital stay, and related factors are an important metric for assessing hospital care, guaranteeing quality, accessibility, and supplementing resources. Despite this inference, I have provided a few remarks and recommendations that the authors should consider before publication in PLOS ONE.		Dear respected reviewer, we thank you for your genuine comments. The comments are helpful to increase the quality our study.

Abstract	- Please ensure that all acronyms are defined on first mention.<br><br>- Better to summarize the background part = make it sound.<br>- on result part Does emergency admission results in longer hospital stay? - the AOR of 0.30, doesn't signify about that, rather it has protective effect.<br>- on conclusion - emphasizing children's cause of admission? what does it mean?? or be specific?		-Thank you! Respected reviewers for your genuine comments, now it has been corrected.<br>- Thank you, revised based on the comment<br><br>-Emergency admission was found to be less likely associated with prolonged stay in the hospital; which means that, those patients presented with non-urgent surgical condition has more likely to stay long time.
Background	- The author concentrated on the unmet need for surgical care in paragraph four of the background section. Does it connect to a hospital stay? If so, it would be better to concentrate on research that shows the percentage of paediatric surgery patients who have a prolonged hospital stay.<br>- The authors indicated in the final paragraph of the background section that limited study was the reason for conducting the study, but this is insufficient. I suggest the authors address the gaps in the study setting and other justifications for conducting the study.		-Thank you, revised….the issue here is if there is unmet need, there is deliance of management; if there is deliance, there is also complication of cases and ultimately increases hospital length of stay even after intervention.<br><br>-okay, thank you! Corrected! See the re-vised manuscript.<br><br>.

Method	- add full stop for the statement of study population.<br>- Measurement for some variables is required. e.g. comorbidity??, prophylaxis medication??, medication??, Anamia, Type of operation, Types of anaesthesia, postoperative complication, blood transfusion, and complexity of the surgical procedure, SA physical status classification,Admission diagnosis, Novel Paediatric Comorbidity, Type of comorbidity (non-chronic disease and Chronic disease), Preoperative anaemia, Nutritional status at admission, Wating time to seek medical attention in hospital, Preoperative Platelet count, Preoperative WBC count, Intra and post-operative<br>characteristics (WHO safe surgical checklist, BUPA scoring, Type of surgery, Prophylaxis, Type<br>of anaesthesia, Duration of anesthesia,Duration of surgery, Postop complication, Type of complication, Blood transfusion, Wound care per day???<br>- ACSH vs ATRH vs AHMC - please indicate all this abbreviation correctly., it seems three hospitals.<br>- the list variables on the data collection section and on the independent variables are not in line.<br>- ASA classification??, Magnitude of the surgery (BUPA scoring)?? how? clearly define and cite it. -HMIS? BSC?? - define at first instant.<br>- MUST?? BMI?? how did you assess and classify it.<br>- I don't think so pre -test was done?? by looking		- Thank you, corrected based on the recommendation and you can see the revised manuscript.<br>- Thank you, the measurement is based on physician diagnosis(which has been already identified from the patient chart) e.g. for those patients having concomitant disease /comorbidity, It has been labeled as ‘yes’ otherwise ‘NO’ and the same is true for other mentioned variables in the comment.<br>- Thank you, corrected based on the recommendation

	all those variables? not defined well.<br>- Prolonged length of hospital stay (PLOS). - define at first instant.<br>- on statistical analysis part the sentence " Statistical significance was determined at a p-value of less than 0.05 with adjusted odds ratio and 95% of CI' was mentioned three times - please avoid redundancy.<br>- please revise the statistical analysis part so as to avoid the redundant sentences.<br>- 100% response rate?? your justification??<br>Result<br>- Error! Reference source not found.) on the Preoperative Characteristics?? please also clearly define some variables listed with abbreviation.<br>- when describing the proportion of sub-categories, please state the magnitude along with percentage. Is not clear about GI disorder, types of comorbidities, and others? for example, malnutrition only 6 out 422 or 12 (comorbidity).		- Thank you, revised based on the recommendation<br>- Thank you, revised based on the recommendation<br>- Thank you, revised based on the recommendation<br>- Thank you, this is because medical charts/patient cards have been replaced by another chart when there is missing of relevant data.<br>- Thank you, revised based on the recommendation<br>- Thank you, revised based on the recommendation
discussion	- all the result part needed through revision and correct the table citations.<br>- The study's reporting and measurement of the length of hospital stay are inconsistent. 75% was the cut-off figure used by the authors to measure the length of hospital stay. However, they reported and discussed with the median. Why? Please modify it.		- Thank you, revised based on the recommendation<br>- Thank you, we have used 75th percentile to determine the cut of value for prolonged length of stay and was found to be >=12.5…. Even though, there is no accepted standard cutoff point, some studies preferred to use 75th percentile due to minimizing procedure specific variations in LOS and possible outliers;,in addition we have also checked the length of stay with median and

	- Nutrition status?? vs comorbidity (malnutrition)? - multicollinearity<br>- urology, and anomaly? on factors table? what does it mean - Urology- the branch of medicine that focuses on surgical and medical diseases of the urinary system and the reproductive organs.?? it's not a disease.<br>- Urology (4) on factor table- < 5, does it violate assumption of chi-square??<br>* The study's discussion, conclusion, limitations, and strengths are all too shallow and require modification. Furthermore, the recommendation is too shallow and should be revised again.<br>422? Is it large?		found to be 6, measure of interest in day; we have discussed peripherally by median as median is the most meaningful comparison measure for evaluating LOS data.<br><br>-Thank you, we have revised it.<br><br>- Thank you, revised<br><br>- Thank you, revised based on the recommendation
Discusion	- What about discussion based on the percentage/magnitude??<br>- What about using variable measurement as a constraint?<br>- limitation on quality of the data?<br>- Complete and thorough revision of the manuscript to remove typos and grammatical errors.		- Thank you, revised based on the recommendation<br>- Thank you, revised based on the recommendation<br>- Thank you, revised<br>- Thank you, it has been now revised based on your recommendation. generally, the comment was found to be help full to increase the quality of this manuscript; thank you once again!
