# Supplementary material for: Prolonged length of stays and its associated factors among pediatric surgical patients in Oromia, Central part of Ethiopia: A cross-sectional study
Source: PLoS One. 2025 Sep 10;20(9):e0330034. doi: 10.1371/journal.pone.0330034 (PMC12422484; doi:10.1371/journal.pone.0330034)
Supplement: Attachment — Submitted filename: point_by_point_response_PLOS, v 2 (1).pdf [file pone.0330034.s003.pdf]

Title: **Prolonged Length of Hospital Stays and its Associated Factors among Pediatric Surgical Patients Admitted in Adama comprehensive specialized hospital and Asella Referral and Teaching Hospital, Oromia, Ethiopia: A cross-sectional study**

From: Authors

To: PLOS ONE

Version: 2

Point -by-point response by authors

Sections	Independent Review Report		Response to comments by Authors
	Reviewer #1		
General comments	Thank you for addressing the comments. All comments from the previous review were addressed except the typographical, grammatical, and punctuation, which needed to be addressed.		Dear respected reviewer, we thank you for your genuine comments. The comments are helpful to increase the quality our study. The typographical, grammatical issue was tried to correct through critical review and software detection.
	Reviewer #2:		
General comments	. I appreciate you inviting me to this manuscript once more, editor. Despite some advancements, the paper still requires development before it can be accepted for publication; for this reason, I have highlighted a few areas that the authors should address below. General comment: In the response letter, the authors didn't provide clear justification for their response or correction for previous comments and suggestions; at least they need to clearly pinpoint the area of amendment.		Dear respected reviewer, we thank you for your genuine comments. The comments are helpful to increase the quality our study.

Abstract	- Is being limited research on this topic in Ethiopia, particularly in the study setting, the only rationale to conduct the study?<br><br>- In the method section, at least incorporate the methods used to assess the outcome variables.<br><br>- Your result indicated the overall length of stay was found to be 19.6%. What about the percentage of prolonged length of stay?<br><br>- Did the emergency admission result in a prolonged length of hospital stay? How? Or at least separate the variables that are positively or negatively related to prolonged length of hospital stay.<br><br>- Conclusion - Approximately one-fifth of the participants experienced prolonged hospital stays vs how about overall length of stay - please make it consistence with the result part of the abstract.		-Thank you!. The study emphasizes on the evidence gap [limited study], geographical gap and gap in the study population (pediatrics). The gap is incorporated and tracked in the attached manuscript.<br><br>- Thank you, revised and tracked based on the comment.<br><br>- Thank you, this is typographical error. The percentage is for PLOS. It is corrected and tracked.<br><br>-Thank you. The emergency admission was negatively related to PLOS. The description [Factors associated] was corrected and tracked according to your suggestion to separate narration.<br><br>-Thank you, corrected. We tried to be consistent with abstract summary [conclusion] and the result. The overall LOS was mistakenly included. Only PLOS was tried to be narrated. Its corrected and tracked.

	- the abstract in the system and on the manuscript are slightly different.<br><br>- recommendation still is not feasible base on the finding, please at least recommend for those variables specifically.\<br><br>- How did you manage healthcare costs? is it your finding?		-Thank you. Adjustment was made in the online system.<br><br>-Thank you, corrected!. Specific recommendation was provided.<br><br>- Thank you. It was an error made in preparing the manuscript. It is removed and tracked.
Introduction	.<br>- Dood improvement- still needs further discussion on the rationale/justification or significance of the study.<br>- Ethiopian context is not well explored.		-Thank you. Some improvement was made in the rationale and some evidences were incorporated in Ethiopian context.<br><br>.
Method	- Why were neonates excluded? any justification? Moreover, on ethical consideration you said" This study did not expose neonates with sepsisto unnecessary risks"? so why if excluded?		- Thank you,this is editorial issue, it has been revised; see the revised manuscript.

	- please provide at least schematic presentation for Sampling procedure? to easily visualize how samples were selected from the two hospitals?<br><br>- Data Collection Methods - Replace with Data collection Techniques and Procedures - and add more on the procedures that were undertaken during the data collection processes.<br><br>- Dependent and independent variables - please summarize the sentence like "Prolonged length of hospital stay is the dependent variable. whereas socio-demographic-related factors (age, sex, residency, religion, and medical insurance); pre-operative factors (comorbidity, prophylaxis medication, medication, and anaemia); and intraoperative and postoperative factors (type of operation, anaesthesia, postoperative complication, blood transfusion, and complexity of the surgical procedure) were the independent variables".<br>- Nutritional status? how did you measure or classify it? please report in the method section and also include in the independent variable list.<br><br>- on outcome variable measurement - 75th percentile for the entire study population were used; it is not clear;		-Thank you, revised based on your comment. See **figure 1**on the manuscrpit<br><br>-- Thank you, revised based on your comment.<br><br>-- Thank you, corrected based on the recommendation<br><br>- Thank you, corrected based on the recommendation. See the revised manuscript.<br><br>-Thank you. Little explanation is provided and tracked in the attached manuscript.

	better to explain so as to not to confuse the readers.<br><br>- the questionnaire was pretested 5% of the sample public hospital (Bekoji hospital). Therefore, what modification wasdone?<br><br>- Does checking normality distribution is appropriate for this data type? may be chi-square distribution? logistic regression assumptions? - please consult biostatisticians.		-Thank you, based on the pretest, some modification was made on the check list…comorbid variables including nutritional status added due to its accessibility on patient medical chart; whereas variables like parental education, occupation, socioeconomic issue and other psychological factors were removed as it was indicated under limitation part of this manuscript.<br><br>-thank you for your insight, it is not as such important based on my consultation and removed accordingly.
Result, discussion&coculusion	- some variables were included in the results part that were not in the independent variables lists -please revise and consider it.<br><br>- On factors associated with PLOS among paediatric surgical patients - please check the chi-square assumptions as one of the cells of the variables have below 5 - or modify the variable classification or		- Thank you, revised based on the recommendation<br><br>- Thank you. We have tried to prevent CI inflation trough cross-tabulation of each cell. To the best of our knowledge, if the rows of a variable are more than 2, one cell

	shorten into two or three levels<br><br>- Discussion is to shallow - needs further explanation and justification for the discrepancy in the result with the previous study -e.g. Especially discuss regarding difference in the hospital set-up, availability of medical personnel (speciality)<br><br>- recommendation still not feasible, please provide as much as feasible recommendations for hospitals and administrator, policy makers based on the findings.<br>- Limitations of the Study - regarding outcome measurement? is the outcome measured similar with previous comparable studies? over estimation or under estimation might occur.<br>- Grammer edition is still required. THank you		with observed value of less than 5 is acceptable.<br><br>-Thank you, we have revised it.<br><br>- Thank you, it has been now revised based on your recommendation. Generally, the comment was found to be help full to increase the quality of this manuscript;<br>-thank you once again for your genuine comments through out!
