# Supplementary material for: Prolonged length of stays and its associated factors among pediatric surgical patients in Oromia, Central part of Ethiopia: A cross-sectional study
Source: PLoS One. 2025 Sep 10;20(9):e0330034. doi: 10.1371/journal.pone.0330034 (PMC12422484; doi:10.1371/journal.pone.0330034)
Supplement: Attachment — Submitted filename: point by point response - V 3.pdf [file pone.0330034.s005.pdf]

Title: **Prolonged Length of Stays and its Associated Factors among Pediatric Surgical Patients Admitted in Oromia, central part of Ethiopia, 2024: A cross-sectional study**

From: Authors

To: PLOS ONE

Version: 3

Point -by-point response by authors

Sections	Independent Review Report	Response to comments by Authors
	Reviewer #1	
General comments	Dear authors,<br>Thank you for the opportunity to review your work once again. The manuscript has shown significant improvement compared to previous drafts. Please find my feedback below:	Dear respected reviewer, we thank you for your genuine comments. The comments are helpful to increase the quality our study.
Title:	• The title of the manuscript is clear and descriptive including the settings and design. However, for concise presentation I would recommend modifying as follows" Prolonged length of stays and its associated factors among pediatric surgical patients in central Ethiopia: a cross-sectional study".	Thank you, for your comments, we have revised based on your comments. See the revised title on the revised manuscript.
Abstract:	○ Background: in this section the detailed description of this study significance and objectives are mentioned. However, the first three lines seems reptation of already existing evidence. The consecutive lines also worth editing as a reptation of ideas. For instance, this statement can be modified as "However, there is limited evidence in Ethiopia, particularly among paediatrics population".	Thank you, for your comments, we have revised based on your comments. See the revised manuscript.

Introduction:	• Detailed description of previous literature, significance and rationale of the study described. However, typographical and grammatical editing may enhance the clarity for the readers. In addition, concise presentation is needed for focused presentation.	Thank you, for your comments, we have revised the typographical and other grammatical issues. see the revised manuscript.
Methods	• Under inclusion and exclusion criteria authors mentioned that the " neonates" were excluded. Does this mean that those neonates who underwent surgery excluded? Explicitly mentioning the rationale for exclusion of neonates may enhance the clarity for the readers.<br>• Sampling: authors mentioned that the simple random sampling was used in the text. However, in the figure 1 "consecutive sampling" was outlined?<br>• Two teaching hospitals were purposively selected from central Ethiopia. However, it was unclear why two hospitals were selected? Also, it would enhance the clarity if the total number of hospitals/teaching hospitals found in the study settings were mentioned to understand the context.	• Neonatal and pediatric surgery differ significantly due to the unique physiological characteristics and vulnerabilities of newborns(neonates) compared to older infants and children<br>• Thank you, for your comments, we have revised it now. see the revised figure.<br>• Thank you, for your comments, the two teaching hospitals have been selected purposely than other non-teaching/non referral hospitals found in Oromia region centeral part of Ethiopia;since they are the only hospitals that can give pediatric surgery services in a little more detail in comparison with other low level hospitals located near the catchment area.

	• Data collection techniques and procedures: Does the incomplete medical records excluded or replaced by the additional sample?<br><br>• Data processing and analysis: The author classified the length of hospital stay based on the 75th percentile, however, there was no justification why 75th percentile used or references from previous literature or guidelines?	• Incomplete medical records were replaced with additional random sample….and it is indicated under the sub section of data collection techniques and procedures of the manuscript.<br>• Thank you, for your comments…all necessary justification and citation have been highlighted under operational definition subsection of the manuscript.
	Reviewer #2:	
General comments	.<br>Dear Editor,<br>I appreciate you asking me to review this work once more. The manuscript has been greatly improved by the authors. However, I have included some of my thoughts and recommendations for the authors to reconsider in order to further enhance the manuscript and prepare it for a worldwide audience:	Dear respected reviewer, we thank you for your genuine comments. The comments are helpful to increase the quality our study.
Affiliation	- On affiliation for the first author—please modify Hawas to Hawassa.	Thank you, we have revised it.
Title	- Better to modify the topic as " Prolonged Length of Hospital Stays and Its Associated Factors Among Pediatric Surgical Patients Admitted to Teaching Hospitals in Central Ethiopia: A Cross-Sectional Study	Thank you, we have revised it.

Abstract	- on an abstract part of the conclussion i recommend the authors to provide feasible recommendation that aimd to improve the service given at the hospitals.<br>- Do the hospitals have equal lengths of time? Is there no difference among them? Are similar interventions recommended to each hospital? What about administration, infrastructure, availability of staff, and medical facility differences among each hospital? This should be clearly identified to have feasible interventions to solve the problem.	-Thank you, we have revised it.<br><br>-The finding indicates nearly comparable length of stay with similar prognostic factors; since both are teaching hospitals serving similar population in terms of characteristics and have nearly comparable capacity in service delivery as well.
Key words	- Keywords: Pediatrics, Length of Hospital stay, Factors, , Surgeryt, , Central Ethiopia (Please correct the commas and the words.)	Thank you, we have corrected.
Introduction	- The introduction section was greatly improved. I recommend at the end of the introduction section to add the significance of the study.	Thank you, for your comments, we have revised based on your comments. See the revised manuscript.
Methods	- On methods, add a full stop to the sentence about the study population.<br>- The authors need to explicitly state the types of data collected. If the data consists entirely of secondary sources or is supplemented by interviews with caregivers or mothers, this should be clearly indicated in the methods section. Additionally, for the secondary data, it is important to explain why a structured questionnaire was used instead of checklists.<br><br>- I check the authors response on the use of 5% pretest, and a modification was made based on that, but on the main document, no modification was made, so I encourage the authors to include those modifications made due to the pretest on the main document.<br>- Rearrange the position of data quality control after the statistical analysis part.	- Thank you, we have added now<br><br>- Thank you, for your comments, we have revised based on your comments. See the revised manuscript.<br><br>- Thank you, for your comments, we have revised based on your comments. See the revised manuscript.<br>- Thank you, for your comments, we have revised based on your

	- Is there any justification to report both the median and mean of length of stay? Did you check symmetry?	comments. See the revised manuscript.<br>-Median is more appropriate due to skewness as a result of time variability
Results and discussion	- Improved results and discussion sections. I recommend that the authors keep consistency and focus on the objectives (prevalence and associated variables)<br>- better to add a section—the practical implication of the study (on discussion or after it)	Thank you, for your comments, we have revised based on your comments
	- In the Limitation section, consider addressing the differences in measurement approaches used across various studies.<br>Some studies referenced the mean or median, while your study utilized the 75th percentile. Does this create a significant gap when comparing the results? It would be beneficial to discuss this issue more thoroughly in the Discussion section.<br>- Please expand your recommendation about possible intervention that would be made to address the problems.<br>- I recommend that the author conduct a comprehensive review of the entire document. There are several inconsistencies in the use of commas, periods, and grammar throughout. Additionally, it would be beneficial to address recommendations that account for local differences among the selected hospitals, such as implementing subgroup analysis.<br>Thank you.	- Thank you, for your comments, we have revised based on your comments<br>- Thank you, for your comments, we have revised based on your comments<br>- Thank you, for your comments, we have revised the inconsistencies in use of comma and other typographical issues; additionally, the finding indicates nearly comparable length of stay with similar prognostic factors; since both are teaching hospitals serving similar population in terms of characteristics and have nearly comparable capacity in service delivery as well.
