# Supplementary material for: Prolonged length of stays and its associated factors among pediatric surgical patients in Oromia, Central part of Ethiopia: A cross-sectional study
Source: PLoS One. 2025 Sep 10;20(9):e0330034. doi: 10.1371/journal.pone.0330034 (PMC12422484; doi:10.1371/journal.pone.0330034)
Supplement: Attachment — Submitted filename: Review report_PLOS ONE.docx [file pone.0330034.s004.docx]

Review report

Dear authors,

Thank you for the opportunity to review your work once again. The manuscript has shown significant improvement compared to previous drafts. Please find my feedback below:

- Title: the title of the manuscript is clear and descriptive including the settings and design. However, for concise presentation I would recommend modifying as follows” Prolonged length of stays and its associated factors among paediatric surgical patients in central Ethiopia: a cross-sectional study”.
- Abstract:
  - Background: in this section the detailed description of this study significance and objectives are mentioned. However, the first three lines seems reptation of already existing evidence. The consecutive lines also worth editing as a reptation of ideas. For instance, this statement can be modified as “However, there is limited evidence in Ethiopia, particularly among paediatrics population”.

Introduction:

- Detailed description of previous literature, significance and rationale of the study described. However, typographical and grammatical editing may enhance the clarity for the readers. In addition, concise presentation is needed for focused presentation.

Methods

- Under inclusion and exclusion criteria authors mentioned that the “ neonates” were excluded. Does this mean that those neonates who underwent surgery excluded? Explicitly mentioning the rationale for exclusion of neonates may enhance the clarity for the readers.
- Sampling: authors mentioned that the simple random sampling was used in the text. However, in the figure 1 “consecutive sampling” was outlined?
- Two teaching hospitals were purposively selected from central Ethiopia. however, it was unclear why two hospitals were selected? Also, it would enhance the clarity if the total number of hospitals/teaching hospitals found in the study settings were mentioned to understand the context.
- Data collection techniques and procedures: Does the incomplete medical records excluded or replaced by the additional sample?
- Data processing and analysis: The author classified the length of hospital stay based on the 75^th^ percentile, However, there was no justification why 75^th^ percentile used or references from previous literature or guidelines?
